# Distribution of some pectic and arabinogalactan protein epitopes during *Solanum lycopersicum* (L.) adventitious root development

**DOI:** 10.1186/s12870-016-0949-3

**Published:** 2017-01-25

**Authors:** Katarzyna Sala, Katarzyna Malarz, Peter W. Barlow, Ewa U. Kurczyńska

**Affiliations:** 1 Department of Cell Biology, Faculty of Biology and Environmental Protection, University of Silesia, 28 Jagiellońska St, 40-032 Katowice, Poland; 2 Department of Organic Chemistry, Institute of Chemistry, University of Silesia, 9 Szkolna St, 40-006 Katowice, Poland; 3 Silesian Center for Education and Interdisciplinary Research, University of Silesia, 75 Pułku Piechoty 1A St, 41-500 Chorzów, Poland; 4 School of Biological Sciences, University of Bristol, 24 Tyndall Avenue, Bristol, BS8 1TQ UK

**Keywords:** Arabinogalactan proteins, Autografting, Cell differentiation, Cell wall, Immunohistochemistry, Lateral roots, Pectins, Tomatoes

## Abstract

**Background:**

The adventitious roots (AR) of plants share the same function as primary and lateral roots (LR), although their development is mainly an adaptive reaction to stress conditions. Regeneration of grafted plants is often accompanied by AR formation thus making the grafting technique a good model for studying AR initiation and development and their means of emergence. Pectins and arabinogalactan proteins (AGP) are helpful markers of particular cellular events, such as programmed cell death (PCD), elongation, proliferation or other differentiation events that accompany AR development. However, little is known about the distribution of pectins and AGPs during AR ontogeny, either in the primordium or stem tissues from which AR arise or their correspondence with these events during LR formation.

**Results:**

AR were developed from different stem tissues such as parenchyma, xylem rays and the cambium, depending on the stem age and treatment (grafting versus cutting) of the parental tissue. Immunochemical analysis of the presence of pectic (LM8, LM19, LM20) and AGP (JIM8, JIM13, JIM16) epitopes in AR and AR-associated tissues showed differential, tissue-specific distributions of these epitopes. Two pectic epitopes (LM19, LM20) were developmentally regulated and the occurrence of the LM8 xylogalacturonan epitope in the root cap of the AR differed from other species described so far. AGP epitopes were abundantly present in the cytoplasmic compartments (mainly the tonoplast) and were correlated with the degree of cell vacuolisation. JIM8 and JIM13 epitopes were detected in the more advanced stages of primordium development, whereas the JIM16 epitope was present from the earliest division events of the initial AR cells. The comparison between AR and LR showed quantitative (AGP,) and qualitative (pectins) differences.

**Conclusion:**

The chemical compositions of adventitious and lateral root cells show differences that correlate with the different origins of these cells. In AR, developmental changes in the distribution of pectins and AGP suggest the turnover of wall compounds. Our data extend the knowledge about the distribution of pectin and AGP during non-embryogenic root development in a species that is important from an agronomic point of view.

## Background

Plant roots have two important functions – to anchor the plant in the soil and to absorb water and mineral nutrients. During normal development, a plant produces a primary root during embryogenesis [1, 2], followed by lateral and adventitious roots, which are formed later during seedling or adult development [1, 3, 4]. AR often develop spontaneously from the above-ground plant parts [5] as a plant’s Bauplan unfolds. However, in some cases AR development is an adaptive reaction of plants to a stress, such as wounding or flooding. AR also form in in vitro cultures of plant parts [6–8] and represent a partial regeneration of the plant from which the cells or tissue were taken. Generally, AR develop from a variety of stem tissues such as the pericycle, the vascular parenchyma, the phloem and cambium [1, 9, 10].

Grafting is widely used in various aspects of plant biological research [11]. It is routinely used in relation to asexual reproduction and is also used for the enhancement of resistance, improvement of quality and to increase the production of agronomically important plants. In recent years, this method has demonstrated an exchange of genetic information between a scion and stock and is thus a useful tool to study horizontal gene transfer [12]. In the specific case of tomato, which is the focus of the present study, grafting is used to improve the quality of tomato cultivars and their tolerance to different environmental factors such as low temperatures and salinity [11, 13–15]. Among the various cellular events that are triggered by grafting, we focused on AR development with particular emphasis on the distribution of the pectic and AGP epitopes during this process in both the AR and the scion tissues surrounding the developing AR.

Homogalacturonan (HG), a major pectin of the dicotyledonous primary cell walls [16], contributes to cell extension, wall porosity and plant defense responses [17]. The structure of HG is built from linear chains of galacturonic acid residues, to which methyl or acetyl groups (methyl- and acetyl-esterification) [18, 19] or other monosaccharides such as xylose or apiose (forming domains known as xylogalacturonan and apiogalacturonan) may be added [18]. The degree of esterification (DE) influences HG properties and can be modified by pectin methylesterases (PMEs) [20]. Moreover, the DE status varies during the life of cells and tissues [21]. Studies of the distribution of HG with different DE have been used to explore the development and senescence of plant structures, the ripening and softening of fruit and vegetable tissues, the effects of fungal infection, as well as in the analysis of various species- or taxa-specific cell wall composition [22–29]. These studies are necessary to understand the cell wall structure and changes in its composition in relation to developmental processes [22].

AGP belong to the hydroxyproline-rich glycoprotein superfamily with a high level of type II arabinogalactan glycosylation [30]. AGP are widespread in plants and their presence has been detected in the cell walls, plasma membranes and in extracellular secretions. Such a ubiquitous presence suggests that AGP are important for plant cell structure and function, as they are. involved in processes such as cell expansion, division and death, seed germination, pollen-tube growth and resistance to infection [30].

Information concerning the chemical composition of root cell walls has come only from studies on primary roots [31, 32]. Studies on sugar beet roots showed a variable distribution of wall epitopes such as JIM7, JIM5, LM6 and LM5, whose expression depended on the stage of root development and the tissue studied [33]. It was shown for several species that the LM8 anti-xylogalacturonan antibody was associated with the detachment of root cap cells [34]. In the case of the primary roots of *Daucus carota, Zea mays, Pisum sativum, Brassica napus, Benicasa hispida, Alnus spp., Oryza sativa* and *Arabidopsis thaliana,* the presence of different pectin and AGP epitopes has been analysed (for detail see [30, 35]). The differential distribution of the AGP epitopes that are recognised by JIM4, JIM13, JIM14 and JIM15 antibodies were described for roots of carrot, pea, radish and onion [32, 36]. Many of the analysed AGP epitopes were specific for root cap cells [37], while others were specific for rhizodermal [38] and elongating cells [39], the root pericycle, the endodermis or young xylem cells [40, 41].

From the data summarised above, it appears that the chemical composition with particular emphasis on the pectic and AGP epitopes of primary roots is well described. In contrast, the different pectic and AGP epitopes in the AR tissues and in the tissues that are penetrated by AR during their development and emergence from parental tissues has not been described to date. Thus, the aims of the study were: 1/to identify any morpho-histological changes that accompany AR development, 2/to analyse the chemical composition of the cell walls of AR and surrounding tissues, 3/to check whether adventitious-derived LR have a distribution pattern that is similar to the pectic and AGP epitopes in AR.

## Methods

### Plant material and sample preparation

Seeds of *Solanum lycopersicon* L. ‘Moneymaker’ were germinated in Petri dishes with wet blotting paper for 5–7 d at 23 ± 1 °C in darkness. Germinated seedlings were transferred to pots with soil and grown at a temperature of 23 ± 1 °C, relative humidity 35% and 16 h photoperiod. 30–40-d-old plants with undamaged cotyledons and an epicotyl length of about 1–1. 5 cm were autografted, as described in [42]. The middle of the epicotyl was cut transversely with a razor blade and the apical part (the scion) was carefully placed and aligned on the basal part (the stock). Toothpicks were used to support the grafted stems and the grafted area was protected by a parafilm tube. In order to prevent excessive wilting, plants were watered and enclosed within a plastic cover to increase the relative humidity for 5 d.

Fragments of grafted stems were collected for analysis during two time periods – from 5 to 10 d and between 20 to 30 d after grafting. Concurrently, control plants of similar ages were cut and put in beakers with tap water to check whether there were any differences between the developing and emerging AR from plants that had been wounded by cutting and the AR that were developing on grafted stems. The latter were designated for further investigations.

Analysis of plants morphology was carried out using an Olympus (Tokyo, Japan) SZH10 stereomicroscope. Surface staining with aqueous solutions of 0. 05% (w/v) Toluidine Blue 0 (Sigma) and 0. 02% (w/v) Ruthenium Red (Sigma) were applied to the hand-cut sections (incubation in dye solution for 10 min, rinsing three times with distilled water).

Fragments of the grafted epicotyls were fixed in a mixture of 3% (w/v) paraformaldehyde (Polysciences), 1. 25% (v/v) glutaraldehyde (Sigma-Aldrich) in phosphate-buffered saline (PBS), pH 7. 2. Samples were de-aerated in fixative for 2 h and incubated in fixative at 4 °C overnight. After rinsing with PBS (three times, 20 min each), the material was dehydrated in an ethanol series (10, 30, 50, 70, 90 and 100%; v/v) and embedded in Steedman’s wax [43]. Longitudinal sections (8 μm thick) were cut using a Zeiss (Jena, Germany) HYRAX M40 rotary microtome and collected on microscopic slides covered with Mayer’s albumin or coated with poly-L-lysine (Menzel Gläser, Germany).

### Histochemistry

Sections were de-waxed, rehydrated in a successive ethanol series (three times in 100%, once in 90% and 50% v/v and then in distilled water, 10 min each wash) and designated for the following histochemical schedules:

### Periodic acid-schiff’s (PAS) method to detect starch, cellulose and carboxylated polysaccharides

Sections were oxidised in a 0. 5% (w/v) aqueous periodic acid (Sigma-Aldrich) solution for 1 h at room temperature, washed in running water for 10 min and rinsed once with distilled water. Next, the slides were placed in Schiff’s reagent (Sigma-Aldrich) for 15 min in darkness, rinsed with distilled water and transferred to a 0. 5% (w/v) sodium sulphite solution for 1–2 min. After washing with running tap water for 5 min, sections were placed in a 0. 5% (w/v) Toluidine Blue 0 aqueous solution for 5 s to visualise the meristematic cells of the developing AR. They were then dehydrated in an ethanol series (20% and 40% ethanol – 2 min each, 60%, 80% and 100% ethanol – 3 min each; v/v) and 100% isopropanol for 3 min. Slides were shaken dry and mounted in Euparal (Roth).

### Sudan III staining to detect lipid substances

Sections were stained with a 0. 5% % (w/v) Sudan III (Sigma) solution (0. 1 g of Sudan III dye was dissolved in 10 ml of 95% ethanol, filtered and 10 ml of anhydrous glycerol (Sigma) was then added and the solution was filtered again). Slides were stained for a minimum 3 h (5 min at temperature of 80 °C and then allowed to cool for 20 min; these steps being repeated several times), rinsed with 50% (v/v) ethanol, rinsed with distilled water and mounted in 50% (v/v) glycerol.

### Aniline blue staining to determine callose localisation

Sections were stained with 0. 1% (w/v) Methyl blue (Sigma) in a 0. 15 M K_2_HPO_4_ solution for 20 min, rinsed three times with distilled water and mounted in 50% (v/v) glycerol.

### Immunocytochemistry

For the immunolabelling procedure, sections were de-waxed and rehydrated in an ethanol series (three times in 100, 90 and 50% in PBS, v/v, each for 10 min). The area occupied by the sections on the microscope slides was marked using a hydrophobic PAP pen (Sigma-Aldrich). The detailed steps of procedure were performed exactly as described by Sala et al [44]. The primary rat monoclonal antibodies (Plant Probes) that were used are listed in Table 1. The secondary antibody that was used was AlexaFluor 488 goat anti-rat (Cat. No. 112-545-003; Jackson ImmunoResearch Laboratories). Sections were stained with 0. 05% (w/v) Toluidine Blue O in PBS for 10 min to quench tissue autofluorescence. Negative controls were performed by omitting the primary antibody step, thereby obtaining no fluorescence signal in the control set of sections.

**Table 1:** List of primary rat monoclonal antibodies used in the current study for the detection of AGP and pectins in adventitious roots

Antibody	Epitope	Reference
*Pectins*		
LM19	HG (non-methyl-esterified, partially methyl-esterified)	[21]
LM20	HG (methyl-esterified)	[21]
LM8	Xylogalacturonan (HG domain)	[34]
*AGPs*		
JIM8	Arabinogalactan	[53]
JIM13	Arabinogalactan/Arabinogalactan protein	[36]
JIM16	Arabinogalactan/Arabinogalactan protein	[36]

All observations and photography were carried out using a Nikon Eclipse Ni-U microscope equipped with a Nikon Digital DS-Fi1-U3 camera with corresponding software (Nikon, Tokyo, Japan), and a maximum excitation wavelength of 490 nm (AlexaFluor 488) or 330 nm (Methyl blue). Photographs and diagrams were edited using the CorelDrawX7 graphics program.

## Results

### Adventitious roots – morphology

**Fig. 1:**
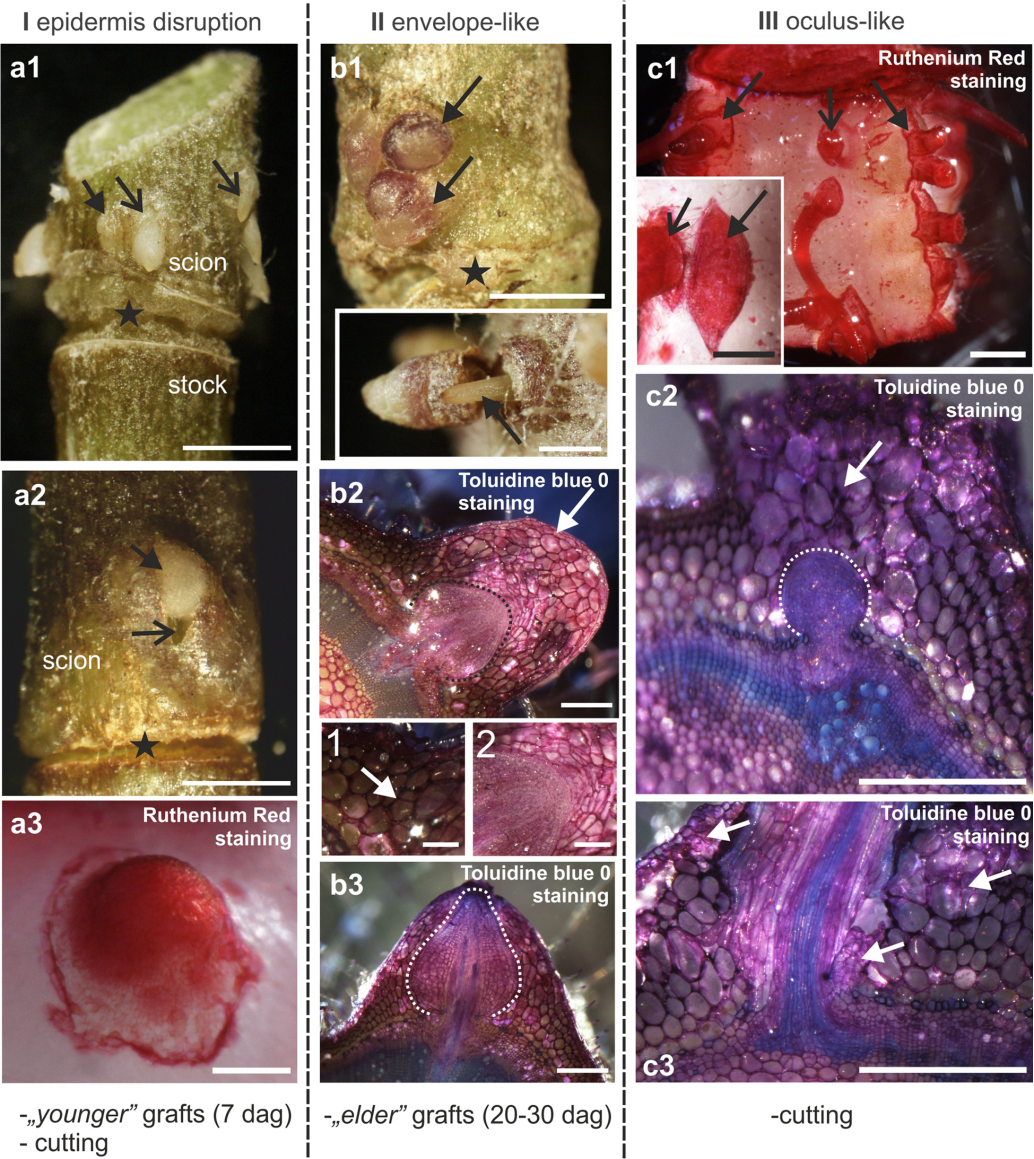
Adventitious roots that formed on tomato stems – morphology and three pathways of their emergence. **a1**-**a3**. Emergence by means of epidermis disruption observed in ‘young’ grafts or cut stems (I). a1. Multiple AR on a scion that had already emerged (*open arrows*) or were about to (*arrow*). a2. An empty space (*open arrow*) is formed between the AR (*arrow*) and the scion as a result of tissue loosening or lysis. a3. AR are covered with pectic substances at the root tip. **b1**-**b3**. Occurrence of an envelope enclosing AR observed on ‘older’ grafts only (II). b1. Envelope surrounds the developing AR primordia (*arrows*) but may be detached (*inset)*, leaving intact roots (*arrow*). b2. Transverse section through an adventitious root (*dotted line*) enclosed within an envelope consisting of large parenchymatous cells (*arrow*); *inset 1*: the envelope originates from scion parenchyma cell division (*arrow*) and further cell proliferation; *inset 2*: root and envelope cells remain in close contact. b3. ARs formed on stems with secondary growth have a specific shape (*dotted line*) at the primordium stage and have a shape that is different from that of the ARs that formed on young stems. **c1**-**c3**. Occurrence of an oculus-like opening observed on cut stems only (III). c1. Oculus-like openings are visible before root emergence (*arrows*); their size is variable and they comprise of one or more emerging ARs (*open arrows*). c2. Openings filled with large, loosely attached callus cells (*arrow*) are established at an early stage of ARs development (primordium outline marked by dotted line). c3. Callus cells are of a parenchymatous origin (*arrows*). asterisk – callus cells, dag – days after grafting. Scale: 2.5 mm (a1, a2), 1 mm (b1, *inset* b1, c1), 0.5 mm (a2, a3, c1 *inset*, c2, c3)

The AR that originate from tomato stems emerge in three different ways (I, II, III), depending on the stage of stem development and the type of procedure that is used to induce their growth (Fig. 1). In the first way, emergence was through epidermis disruption (Fig. 1 a1, a2, a3) and took place in cut stems or ‘young’ scions approx. 5–7 d after the graft was prepared and when the secondary growth of stem had not yet commenced. The cortex and collenchyma had also become disrupted and loosened (Fig. 1 a2). The surface of emergent roots was covered by pectic substances (Fig. 1 a3). In the second way, the scion tissues formed an envelope-like structure around the new root (Fig, 1 b1), which eventually detached (Fig. 1 b1, *inset*). The ‘envelope’ consisted of parenchymatous cells which originated from the proliferation of the collenchyma (Fig. 1 b2 and *inset 1*). The root and these parenchymatous cells remained in close contact during the early stage of root growth (Fig. 1 b2, *inset 2*). The formation of the “envelope” occurred in older grafts, i.e. at approx. 20 d or more after grafting, when the stems were exhibiting secondary growth. The AR that were surrounded by this parenchymatous ‘envelope’ had a bulbous outline (Fig. 1 b2 and b3). In the third way, which was specific to cut stems only, numerous AR emerged through eye-like openings that had formed on the stem and were filled with friable cells (Fig. 1 c1). The epidermis became disrupted and large isodiametric cells protruded from the stem (Fig. 1 c2). Even when the primordium was still at a relatively early stage of growth, the opening was already established (Fig. 1 c2). The cells filling the opening originated from dedifferentiated collenchyma cells, but sub-epidermis and cambium, through their cell division, contributed as well (Fig. 1 c3).

### Adventitious roots – histological type I and II

**Fig. 2:**
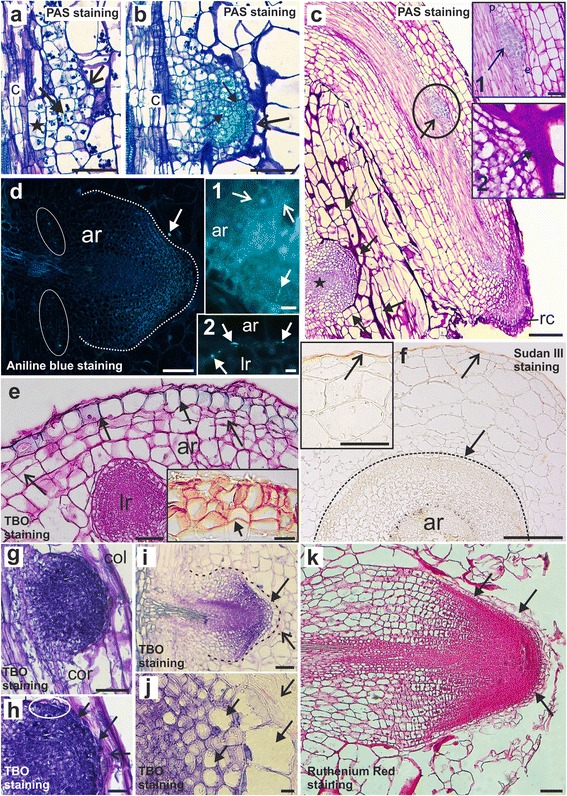
Histology of adventitious roots from tomato-grafted stems. **a** Division of the initial cells (*arrow*) and thickened walls at the AR/scion tissue border (*open arrow*); asterisk – multicellular complexes. **b** Meristematic cells of a young primordium (*arrows*) and scion cells (*open arrow*). **c** AR bearing LR initials (*open arrow*) and cell divisions in the parenchyma (*arrows*) next to a developing AR (*asterisk*); *inset 1*: LR pericycle origin (*open arrow*); *inset 2*: thick layer between the AR and scion (*arrow*). **d** Callose in the phloem (*ellipse*) and the layer between the AR (*dotted line*) and scion (*arrow*); *inset 1*: callose in the primary pit fields (*open arrows*) and cell plate (*arrow*); *inset 2*: callose (*arrows*) between the AR/LR border. **e** Polyphenols in the rhizodermis (*arrows*) and periclinal cell divisions beneath (*open arrows*); *inset*: older AR, lipids in the phellem (*arrow*). **f** Transverse section, AR (*dotted line*) enclosed within the envelope covered by lipid substances (*open arrow*), no lipids in the rhizodermis (*arrow*); *inset*: envelope cuticle (*open arrow*). **g**, **h** AR emerging by means of epidermis disruption; the root cap file starts at the edge of the primordium (circled area) and comprises 1 to 2 cell files (*arrows*), adjacent to layer of dead cells (*open arrow*). **i** AR primordium (*dotted line*) emerging within an envelope; root cap cells (*arrow*) adjacent to a layer of dead cells (*open arrow*). **j** Root cap cells of various sizes (*arrows*) next to dead cells (*open arrow*). **k** Root cap cells (*arrows*) after emergence. ar – adventitious root, c – cambium, col – collenchyma, cor – cortex, e – endodermis, lr – lateral root, p – pericycle, rc – root cap. Scale: 100 μm (**c**, **d**, **e**, **g**, **i**, **k**), 50 μm (**a**, **b**, **f**, **h**), 25 μm (**f** *inset*); 10 μm (**c** *inset 1* and *2*, **d** *inset 1 and 2*, **e** *inset* and **j**)

At the earliest stage of AR development, initial cells, which were rich in starch, showed cell divisions, thereby forming multicellular complexes (Fig. 2a). The cell walls became thickened at the boundaries between the developing AR and the scion tissues (Fig. 2a). The root primordium consisted of actively dividing meristematic cells, each having a dense cytoplasm, a large nucleus and reduced starch content (Fig. 2b). As development proceeded, the boundaries between the root and scion cells were composed mainly of insoluble polysaccharides, as was revealed by a PAS reaction, and became thicker (Fig. 2b and 2c, *inset 2*). Within a given scion, multiple AR at different stages of development were found (Fig. 2c). Moreover, LR formed from the pericycle of the AR (Fig. 2c, *inset 1*), independently of the point of emergence of the latter roots. There was no significant occurrence of callose during AR or LR emergence. Callose was present, however, in some areas within the thick layer between the AR and the scion tissues (Fig. 2d), or at the border between the AR and LR (Fig. 2d, *inset 2*). In the AR, callose was detected in the primary pit fields and cell plates of dividing cells (Fig. 2d, *inset 1*). When the AR or LR were still enclosed within the maternal tissues, no lipid substances were detected in their cell walls (data not shown). After root emergence, deposition of polyphenolics in the rhizodermal walls commenced and cells beneath the rhizodermis divided periclinally (Fig. 2e) giving rise to the periderm (Fig. 2e, *inset*). Identification of this tissue was based on its phenotypic features such as the presence of lipid substances within the cell wall, the presence of polyphenols and cell shape.

When the developing AR were surrounded by an envelope of parenchyma, lipid substances were detected only in the cuticle, and were deposited on the outer periclinal cell walls of the so-called “envelope” (Fig. 2e and the *inset*). Cells of a root cap could already be distinguished within the dome-shaped primordium (Fig. 2g), which was formed of two or more cell layers (Fig. 2h). In roots that were emerging within their “envelope”, the outermost root cells were of various sizes and shapes (Fig. 2i and j). Although the boundary between the emerging root and scion tissues was marked by a thickened polysaccharide layer, the outermost root cap cells resembled the cortical cells of the scion (Fig. 2i). After emergence, the root cap remained attached to the root tip (Fig. 2k).

### Adventitious roots – distribution of pectic epitopes

**Fig. 3:**
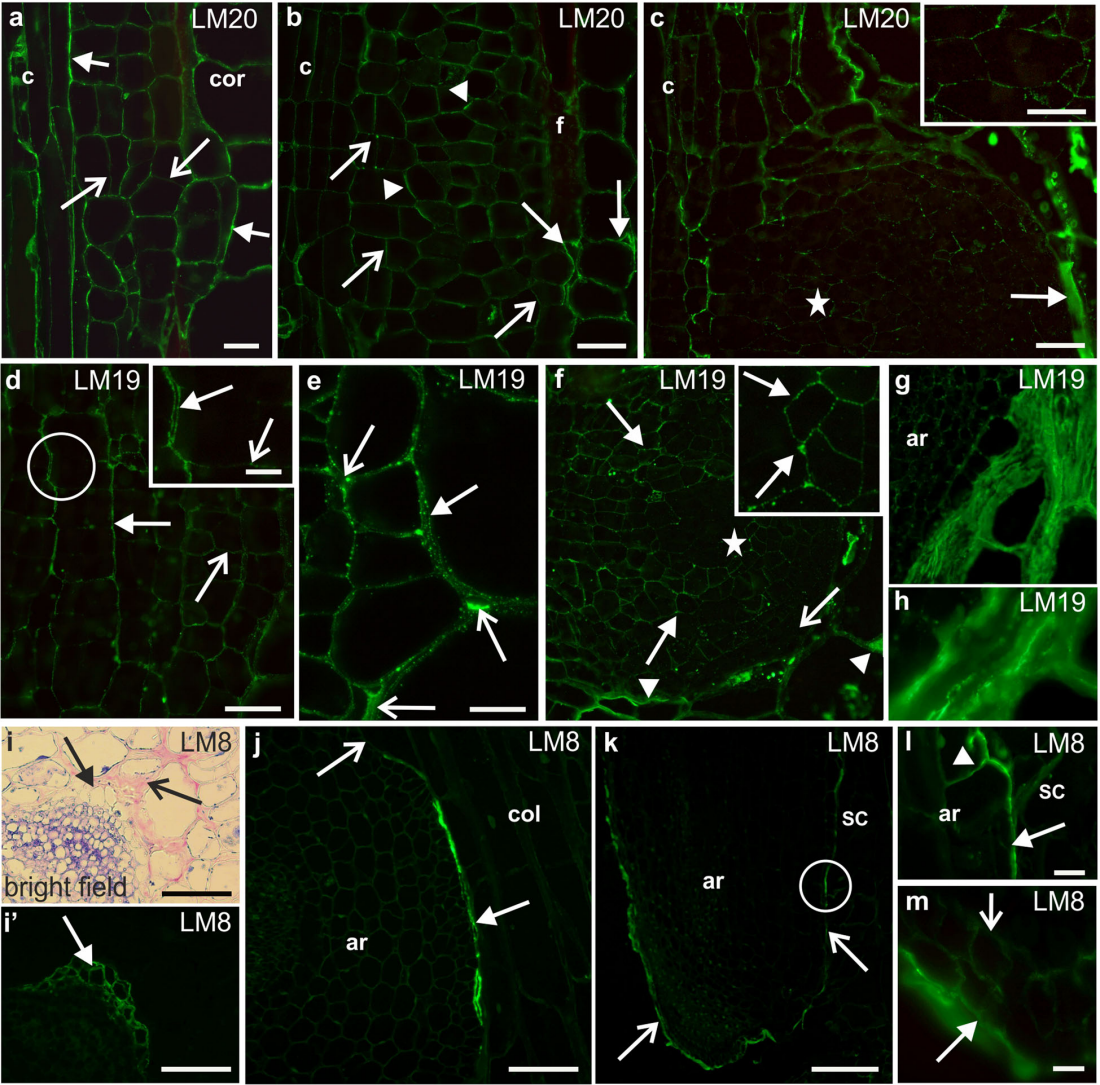
Immunolabelling of adventitious roots during development – pectin epitopes. **a** Initial stage, LM20 epitope in walls between the cambium/initial cells/cortex (*arrows*), weak signal in division walls (*open arrows*). **b** Punctate (*open arrows*) or continuous (*arrowheads*) distribution as divisions proceed, abundant occurrence at AR/cortex boundary, in the walls and intercellular spaces of cortex (arrows). **c** LM20 present in the extracellular layer (*arrow*); *inset*: magnification of c (*asterisk*), weak signal in primordium. **d** Initial stage, LM19 epitope present in initials (*open arrow*) and multicellular complex (*arrow*); *inset*: magnification of d (*circle*), weak signal in the division walls (*open arrow*) in contrast to the walls of the cell complex (*arrow*). **e** Cortex adjacent to a developing root, LM19 localised in the walls, middle lamellae (*arrow*) and intercellular spaces (*open arrow*). **f** LM19 epitope abundant in the ground tissue (*arrows*) in contrast to the central region (*asterisk*) and epidermis (*open arrow*), strong labelling in the cortex walls, intercellular spaces and the extracellular layer (*arrowheads*); *inset*: magnification of f (*asterisk*), epitope distribution (*arrows*). **g**, **h** the extracellular layer. **i**. Parenchyma next to an LR primordium (*arrow*) and thickening of the extracellular matrix (open arrow). i’. **i** fluorescence, LM8 epitope present in the cortical cells adjacent to a primordium (*arrow*). **j** LM8 between the root and scion (*arrow*), no fluorescence signal in sections without contact (open arrow). **k** LM8 in the rhizodermis (*arrows*). **l** Magnification of k (*circle*), LM8 detected in the outer periclinal walls (*arrow*) and a weak signal in the anticlinal walls (*arrowhead*). **m** Root cap, LM8 present in the outer periclinal walls (*arrow*), weak signal in the inner cells (*open arrow*). ar – adventitious root, c – cambium, col – collenchyma, cor – cortex, f – fibre, rc – root cap, sc – scion. Scale: 100 μm(k), 50 μm (**f**, **i**, **j**, *inset* i), 20 μm (**a**-**e**, **g**, **l**, **c** *inset*, **d**), 10 μm (**h**, **m**, **f** *inset*)

At an early stage of AR development, a pectic epitope that was recognised by the LM20 antibody (high methyl-esterified HG) was detected in the walls of all of the initial cells (Fig 3a). However, the fluorescence signal was most intense at the border between the cambium/AR and the AR/cortex of the scion, thus suggesting a higher amount of the epitope in these regions. In contrast, in the newly formed cell walls, the occurrence of the LM20 epitope was hardly detectable (Fig. 3a). As AR growth proceeded, the LM20 epitope was still detected in the initial cells but its distribution was not uniform – in some walls, the signal was more or less punctate, in other walls, its distribution was continuous within the wall and had an intense fluorescence signal (Fig. 3b). The LM20 epitope occurred abundantly in the layer between the AR and the cortex cells of the scion; it was also detected in the periclinal walls of the root epidermis as well as in the periclinal walls and intercellular spaces of the cortical cells (Fig. 3b). In the walls of the initial cells of older AR primordial, the LM20 epitope was abundant and distributed within the wall in a continuous manner. This was in contrast to the other cells of the primordium where the fluorescence signal relating to this epitope was weak and was distributed in a more or less punctate manner (Fig. 3c). The LM20 epitope also occurred in a thick extracellular layer that was formed between the cells of the developing AR and the cortex of the scion (Fig. 3c).

Early in AR development, the pectic epitope that was recognised by the LM19 antibody (low methyl-esterified HG) was less abundant than the LM20 epitope and was distributed in a punctate manner in the walls of the initial cells (Fig. 3d). In the walls of the multicellular complexes, which often accompanied the initiation of the primordium, the LM19 epitope was distributed in the primary walls, but was absent in the newly formed cell walls within a complex (Fig. 3d, *inset*). In the walls of the cortical cells, which were adjacent to a developing root, the LM19 epitope occurred abundantly and was distributed in a punctate manner throughout the primary walls and the middle lamellae; it was also detected in the intercellular spaces (Fig. 3e). In older primordia, the LM19 epitope distribution was more widespread than the LM20 epitope (Fig. 3f). The LM19 epitope was detected in the walls of the differentiating cells in the ground tissue of the primordium, in contrast to its central region and epidermis, from which this epitope was absent (Fig. 3f). In between the AR and the cortex, there was a thick extracellular layer where the LM19 epitope was especially abundant (Fig. 3g); however, it was not the only component (Fig. 3h).

In the AR that developed within the scion tissues, the pectic epitope that was recognised by the LM8 antibody (xylogalacturonan) occurred only in the walls of root cap cells, which were adjacent to the primordium (Fig. 3i and i’). In the part of the mature AR that was enclosed within the scion, the LM8 epitope was detected in the layer between the rhizodermis and the adjacent scion cells (Fig. 3j). Its distribution was not uniform, and the signal was most intense where the contact between the AR and the scion was closest, but was absent from sections where the contact was not so tight (Fig. 3j). In the AR that had emerged, the LM8 epitope was detected in the cell walls of the epidermis. Here, however, its distribution was discontinuous (Fig. 3k) and its occurrence was largely restricted to the outer periclinal cell walls; a weak fluorescence signal was also detected in the anticlinal walls (Fig. 3l). Within the root cap, only small amounts of the LM8 epitope were detected in the cell walls, in contrast to the larger amounts that were observed in the outer periclinal cell walls of the root as a whole (Fig. 3m).

### Adventitious roots – distribution of AGP epitopes

**Fig. 4:**
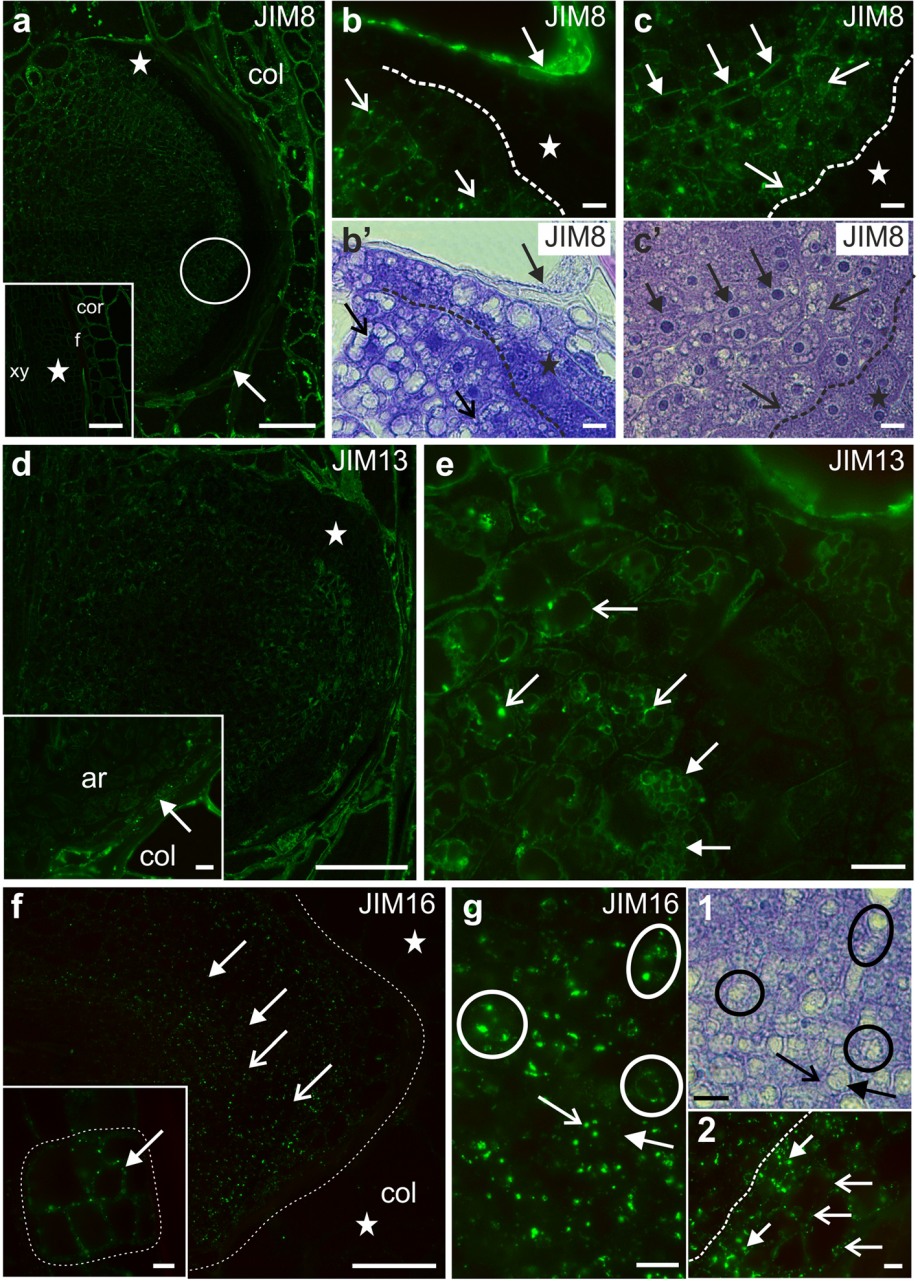
Immunolabelling of adventitious roots during development – AGP epitopes. **a** Primordium stage of AR, JIM8 epitope occurrence in ground and vascular tissue and in the layer between the AR and the scion (*arrow*); *inset:* absence of the JIM8 epitope during the initial stages of AR development (*asterisk*). **b** Magnification of a (*asterisk*)., Abundant occurrence of the JIM8 epitope in the scion cells (*arrow*); increase of the JIM8 presence in particular AR cells (*open arrows*), its absence from root cap and meristematic cells (*asterisk*). b’. Bright field of **b**. **c** Magnification of a (*circled area*), the JIM8 epitope is either localised in the wall (*arrows*), cytoplasm (*open arrows*) or is absent (*asterisk*), depending on the cell file. c’. Bright field of **c**. **d** Primordium stage of AR with the JIM13 epitope occurrence in the ground tissue and some root cap cells; *inset*: the JIM13 epitope present in the layer between the AR and the scion (*arrow*). **e** Magnification of d (*asterisk*). Tonoplast localisation of the JIM13 epitope (*open arrows*). **f** Punctate distribution of the JIM16 epitope in the cells of an AR root primordium (*dotted line*), no signal in scion tissues (*asterisks*); *inset:* the JIM16 epitope is present at the division stage of the initial cells with localisation in walls/plasmalemma (*arrow*) of the complex (*outlined area*). **g** the JIM16 epitope presence in the tonoplast (*circled areas*) and in the cytoplasm (*open arrow*) next to the vacuole (*arrow*);vacuole *inset 1*: bright field of **g**; *inset 2*: abundant JIM16 epitope occurrence in the root cap cells (*open arrows*) and in the meristematic cell files beneath (arrows), the right side of the dotted line. f – fibre, col – collenchyma, cor – cortex, xy – xylem vessels. Scale: 100 μm (**a**, **a** *inset*, **d**, **f**), 10 μm (**b**, b’, **c**, c’, **d** *inset*, **e**, **f** *inset*, **g**, **g** *inset 1 and 2*)

At the earliest stages of AR development, the AGP epitope that was recognised by the JIM8 antibody was not detected in the initial cells or during their subsequent divisions (Fig. 4a *inset*). The JIM8 epitope did appear, however, at the root primordium stage, though here it occurred less abundantly than it did in the scion collenchyma or cortical cells (Fig. 4a and b). JIM8 was not observed in meristematic cells near the border of the root nor in the root cap cells within the primordium (Fig. 4a, b and c); however, it was present in cells from the other files, and here the level of the JIM8 epitope increased parallel with the level of cell vacuolisation (Fig. 4b and b’). The epitope was present in the cell wall and/or plasma membrane (Fig. 4b) as well as in the cytoplasm (Fig. 4c). There was a difference in epitope distribution – from the cell wall/plasma membrane to the cytoplasm – between cells of different cell files (Fig. 4c). The JIM8 antibody signal was also detected in the layer between the emerging root and the scion cells (Fig. 4a).

The AGP epitope that was recognised by JIM13 antibody showed a temporal and spatial distribution similar to that of the JIM8 epitope, with one exception; it did occur in some root cap cells (Fig. 4d). There was also a correlation between the increase in the JIM13 epitope and the level of cell vacuolisation but, in contrast to JIM8, the JIM13 epitope was mainly present in the tonoplast, as well as in the large and small vacuoles (Fig. 4e).

At the earlier stage of AR development, the AGP epitope that was recognised by the JIM16 antibody was detected in the walls/plasma membrane of the dividing initial cells (Fig. 4f *inset*) as a punctate signal and this type of distribution was displayed throughout the entire root development. At the primordium stage, the JIM16 epitope occurred in all of the cells of the developing root with the exception of the future vasculature. This was in contrast to the scion tissues, collenchyma and cortex, where it was hardly present (Fig. 4f). In contrast to the JIM8 and JIM13 epitopes, the JIM16 epitope occurred in the root cap cells and in the highly meristematic cell file next to the root cap (Fig. 4g *inset 2*). The occurrence of the JIM16 epitope was associated with the tonoplast, cytoplasm and plasma membrane (Fig. 4g, *inset 1* and *2*).

### Lateral roots – distribution of pectic and AGP epitopes

**Fig. 5:**
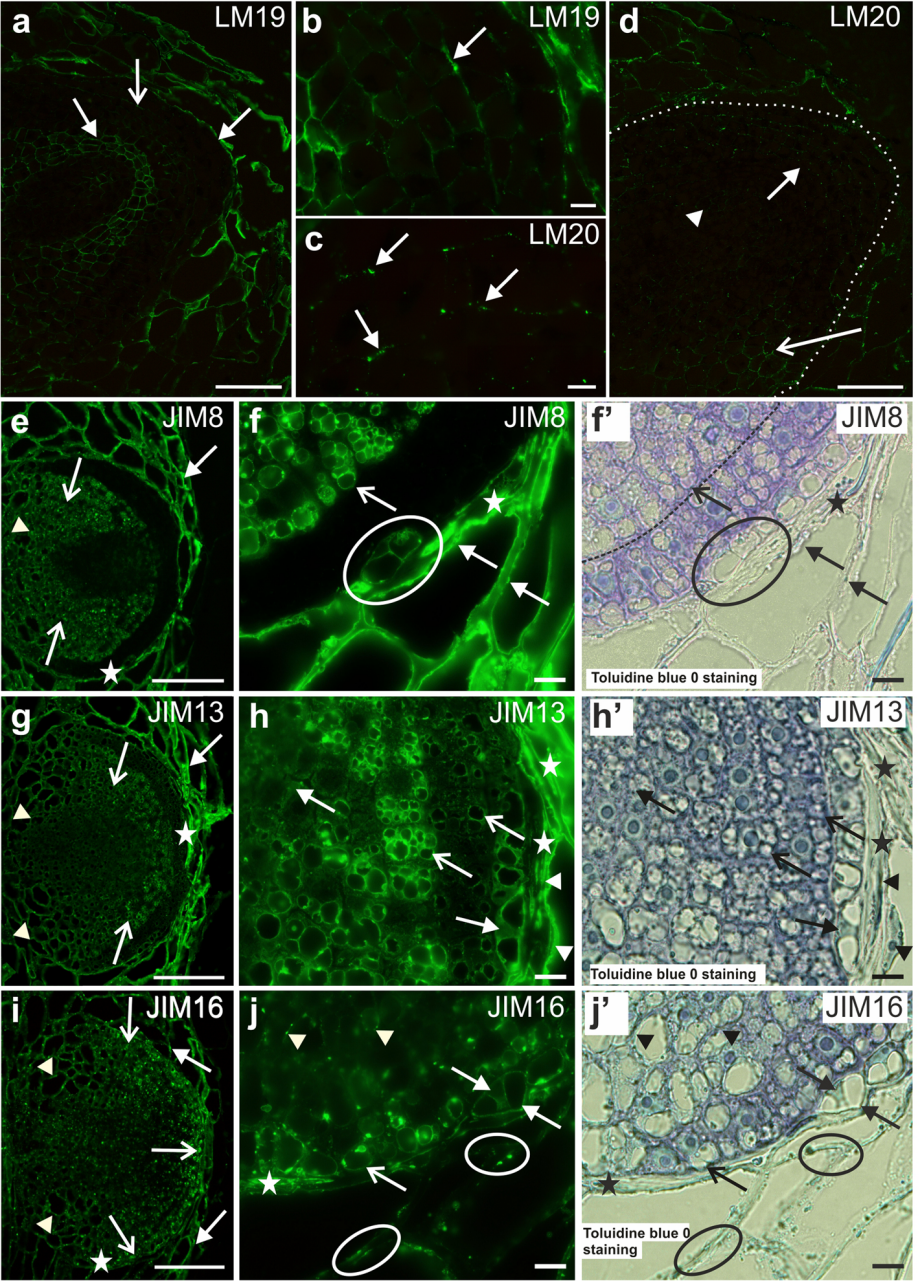
Immunolabelling of lateral roots upon emergence – pectic and AGP epitopes. **a** Localisation of LM19 in the cells adjacent to the future vascular tissue and in the outer periclinal walls of rhizodermis (*arrows*), weak signal in other tissues (*open arrow*). **b** LM19 at the border of root cap and other cells (*arrow*). **c** Punctate signal of LM20 (*arrows*). **d** Low (*arrow*) or no occurrence (*arrowhead*) of the LM20 epitope in LR (*dotted line*) and presence in the ground tissue (*open arrow*). **e** Occurrence of JIM8 in the AR cells (*arrow*) and ground tissue of an LR (*open arrows, arrowhead*). **f** Magnification of e (*asterisk*), tonoplast localisation of JIM8 in the ground tissue (*open arrow*) and some epidermal cells (*ellipse*). In AR, occurrence in the cortex walls and/or plasmalemma (*arrows*) and in the extracellular layer (*asterisk*). f’. Bright field of **f**, vacuolisation degrees of epidermal (*ellipse*), meristematic and ground tissue cells (separated by the dotted line). **g** the JIM13 epitope in the AR cells (*arrow*) and ground tissue of the LR; wall distribution in differentiated cells (*arrowheads*) and cytoplasmic localisation in meristematic cells (*open arrows*). **h** Magnification of g (*asterisk*), JIM13 in cytoplasm (*arrow*), tonoplast (*open arrows*), plasmalemma and/or cell wall (*arrowheads*), abundant occurrence in remnants of collapsed cells (*asterisks*). h’. Bright field of **h**. **i** the JIM16 epitope in AR (*arrows*) and LR cells with an abundant cytoplasmic distribution on the periphery of a primordium (*open arrows*) in contrast to wall localisation in the ground tissue (*arrowheads*). **j** Magnification of i (*asterisk*), continuous (*open arrow*) or punctate (*arrowheads*) tonoplast localisation of JIM16. In the LR epidermis, epitope detected in the plasmalemma, cytoplasm (*arrows*) and in the extracellular layer (*asterisk*); in AR punctate distribution in plasmalemma and/or wall (*ellipse*). j’. Bright field of **j**. Scale: 100 μm (**a**, **d**, **e**, **g**, **i**), 10 μm (**b**, **c**, **f**, f’, **h**, h’, **j**, j’)

Upon the emergence of the LR, the LM19 epitope was found in the walls of the cells enclosing the future vascular tissue and in the outer periclinal walls of the rhizodermis (in sections where the primordium was already separated from the parent adventitious root tissue) (Fig. 5a). In the other cells of the primordium, the LM19 epitope was weakly expressed and was distributed in either a punctuate or a continuous manner (Fig. 5b). The signal of the LM20 antibody was, however, sporadically punctuate (Fig. 5c). The LM20 epitope was hardly present in the primordium with the exception of some ground tissue cells (Fig. 5d). The LM8 epitope was absent in the lateral root primordium (data not shown).

The AGP epitope that was recognised by the JIM8 antibody occurred abundantly in the AR tissue (Fig. 5e). Within the lateral root, however, a complete absence of JIM8 epitope was observed in the cells that were localised on the periphery and in the central region of the primordium zones that corresponded to the future epidermis, stele and root apical meristem. This contrasted with the ground tissue where JIM8 occurred abundantly (Fig. 5e). In the differentiated ground tissue cells at the base of the LR primordium, the JIM8 epitope was present in the cell walls (Fig. 5e). The distribution of the JIM8 epitope within the other cells of ground tissue that had meristematic features (a large nucleus, dense cytoplasm and small vacuoles) was restricted to the tonoplast (Fig. 5f and f’). Within the layer of the rhizodermis, the JIM8 epitope was detected only in the tonoplast of some cells (Fig. 5f and f’). In the cells adjacent to a protruding LR primordium, the JIM8 epitope signal was observed in the plasma membrane and/or primary cell walls, as well as in the thick layer between the borders of the lateral roots and AR (Fig. 5f and f’).

An arabinogalactan protein epitope that was recognised by the JIM13 antibody was observed in similar regions of the lateral root primordia (Fig. 5g), and also occurred in the tonoplast and cell cytoplasm (Fig. 5h and h’). In contrast to JIM8, the JIM13 epitope was also present in some of the meristematic cells at the developing root tip (Fig. 5h and h’). The remnants of cortical cells that had collapsed as a result of the emergence of the lateral root were also rich in the JIM13 epitope (Fig. 5h and h’).

The arabinogalactan protein epitope that was recognised the by JIM16 antibody was detected in all of the tissues of the developing lateral root, either as a continuous or punctuate signal, although in the central region of the primordium, the fluorescence signal was punctate and mostly weak (Fig. 5i). In contrast to the JIM8 and JIM13 epitopes, the JIM16 epitope occurred abundantly in the meristematic cells at the root tip, where it was detected in the tonoplast (Fig. 5j and j’). In.the rhizodermis, the epitope was detected in the cytoplasmic compartments and in the plasma membrane (Fig. 5j and j’). In the AR cells, the occurrence of the JIM16 epitope was observed less easily than the JIM8 and JIM13 epitopes (Fig. 5j).

## Discussion

**Fig. 6:**
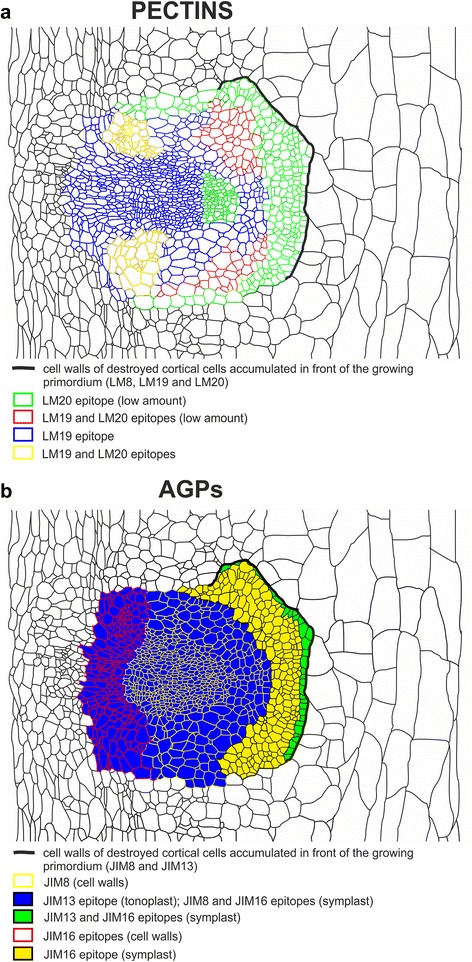
Schematic representation of **a** pectic and **b** AGP epitope distribution in tissues of the primordium (longitudinal section)

The distribution of the LM19 and LM20 epitopes during AR primordium development indicates that these epitopes have distinctive localisations at the corresponding developmental stages in both the developing AR and the scion. At the earliest phase of AR development, the LM20 epitope occurred abundantly, while at a more advanced stage, its presence decreased and its distribution became less uniform. Concurrently, the LM19 epitope increased and, at the primordium stage, it was more widespread than the LM20 epitope. Similarities in the distribution also concerned the AR/cortex border where both epitopes were abundantly detected in a thick layer, whereas neither occurred, or were only faintly detected, in the new cell walls of the divided cells. This is consistent with observations that meristematic cells have methyl-esterified HG domains whereas differentiating cells exhibit more non-methyl-esterified domains [20, 45]. A distinct distribution of methyl- and/or non-methyl-esterified HG was also reported during the somatic embryogenesis of various plants [44, 46, 47] and the embryogenesis of the fern *Ceratopteris richardii* [48, 49] who used JIM5 and JIM7 antibodies, showed that methyl- and un-esterified pectins were located at the interface between the mother and emerging LR of onion. It was shown that these same antibodies strongly labelled the space that was created between the primordium and the main root cells, thus indicating that a release and/or accumulation of pectic fragments occurred at this interface. The results presented here are in accordance with the findings described above because the JIM5 and JIM7 antibodies display some similarities to LM19 and LM20 antibodies [21], which were used in our work. LM19 and LM20 antibodies, as well as JIM5 and JIM7, share epitopes that have a partial degree of esterification [21]. Differences and similarities in the LM19 and LM20 epitope distribution at the primordium stage of development are schematically depicted in Fig. 6a.

Xylogalacturonan, which is represented by the LM8 epitope, was present in the root cap cells of AR during the primordium stage but not at a more advanced stage of development. This epitope is thought to be specifically associated with the root cap cells in a range of angiosperm species [34], thus indicating that roots that have a different origin have the same root cap markers. It must be noted that, in our system, during more advanced stages of AR development, the root cap cells were marked only in the outer pericinal walls, in contrast to the cytoplasmic and wall distribution in the roots of *Pisum sativum*, *Daucus carota*, *Zea mays* and *Arabidopsis* [34]. The LM8 epitope is not only a marker of root cap cells, but is also specifically associated with the separation process that results in complete cap cell detachment [34]. The occurrence of xylogalacturonan was observed during organogenesis in an in vitro culture of wheat, where it was present in detached or loosely connected callus cells [50] and also at the surface of loosely attached cells in embryogenic carrot suspension cell cultures [34]. Moreover, the LM8 epitope was detected in the inner parenchyma cells that are loosened and subsequently crushed by the growing cotyledons during pea testa development [51]. It is obvious that during the development and emergence of AR, the cell walls of the cortical cells are destroyed and pile up as the root primordium advances through the cortical tissue [5], thus forming a thick layer between the root and maternal tissues. In our model, the cortex, collenchyma and epidermis are the tissues through which AR has to protrude – yet, we found no LM8 epitopes in the scion cells surrounding the developing AR. That observation indicates that root emergence is not accompanied by any significant cell detachment.

In the case of the AGP epitopes, it appeared that their presence and distribution depended on the stage of AR development and the cell type. JIM8 and JIM13 antibodies were detected in the more advanced primordium stage of AR development, whereas the JIM16 epitope was present from the earliest division events.

Results from various studies concerning the presence of AGP epitopes in the roots of a range of species are listed in [30, 35]. JIM13 and JIM8 epitopes were found in the border and border-like cells of the root caps of *Arabidopsis thaliana*, *Pisum sativum* and *Brassica napus* [35, 52], whereas JIM8 and JIM16 occurred in the elongating cells of *Arabidopsis thaliana* [39] and in the root apical meristem of *Daucus carota* [36]. In our work, the distribution patterns of the JIM8 and JIM13 epitopes were similar – both appeared at the advanced developmental stage but were absent from the meristematic cell files of the primordium; their amounts increased simultaneously with the degree of cell vacuolisation and finally, JIM8 and JIM13 epitopes were less abundant in AR cells compared to the parenchymatous cells of the scion. The JIM16 epitope, however, was detected at the earliest developmental stages, when the divisions of the initial cells commenced. By contrast, it was hardly present in the scion tissues. Later, the JIM16 epitope occurred in all of the cells of primordia, especially in the root cap and meristematic cell files. Although these results may indicate differences between various species or between the type of roots (primary *versus* adventitious roots), they still point to the important role of AGP in root tissue development.

*In Brasica napus* flowers, a temporal and spatial regulation of the plasma membrane AGP epitope that is recognised by the JIM8 antibody was postulated [53]. During embryogenesis, the JIM8 epitope was at first expressed in the cells of the proembryo, disappeared from the embryo proper but remained in the suspensor. During the differentiation of the stamens and carpels, the JIM8 occurrence exhibited a temporal sequence of distribution as cell differentiation progressed [53]. This indicates that the JIM8 epitope specifies positionally defined tissues or cell types, just like the other AGP epitopes JIM4 [54] or JIM13 [32, 36, 41] in other species or organs. Thus, it was suggested that AGP may act as cell-position marker and convey information that is essential for cell patterning or the establishment of symmetry [36]. Although all three antibodies that were used, JIM8, JIM13 and JIM16, labelled some cytoplasmic compartments with the most evident localisation in the tonoplast, only the JIM16 epitope was present in the meristematic cell files next to the root cap. JIM8 and JIM13 epitopes appeared in those vacuolated cells that were differentiating. This observation may suggest that these AGP can mark cells before any phenotypic changes of cell differentiation are visible [55]. Therefore, our results may point to characteristic AGP expression pattern that may be involved in adventitious or lateral root development.

The distribution of all of the AGP epitopes that were applied within primordium in the presented study is schematically presented in Fig. 6b.

### Comparison of AR to LR

The comparison of LR and AR at the corresponding stages of development showed some differences as well as similarities in the occurrence of the pectic and AGP epitopes. No xylogalacturonan was detected in the LR; moreover, the LM20 epitope (HG, partially methyl-esterified) was hardly present in LR in comparison to AR. The LM19 epitope (HG, partially un-esterified) occurred in the outer periclinal cell walls of the primordium and was especially abundantly in the regions where LR had already emerged. AGP epitopes had a similar distribution pattern in both types of roots. However, they were more expressed in the LR than in the AR. AR share the same function as LR but develop from aerial tissues [6]. The partial similarities in cell components that were observed can be explained in two ways – it is either the origin that influences the cell composition or it is the environment surrounding the developing primordial that is the influence. More studies should be conducted to verify these two assumptions.

## Conclusion

We performed histo- and immunohistochemical analyses of the cell wall to study the development of tomato AR. The AR that formed on the grafted stems differed from the AR that were induced by stem cutting in terms of the shape of the primordium and the way they emerged. Two HG epitopes, LM19 and LM20, had different localisations at the corresponding AR developmental stages with an increase in LM19 occurrence along with the progress of AR growth. Of the three AGP epitopes that were applied, JIM8 and JIM13 showed an association with the tonoplast in particular cells of the primordium, whereas the JIM16 epitope occurred from the earliest stage, and therefore it can be considered to be a marker of initial cells. Finally, the differences observed between the AR and LR cell wall composition might be origin- or environment-dependent.

## Abbreviations

AGPs: Arabinogalactan proteins
AR/ar: Adventitious root(s)
c: Cambium
col: Collenchyma
cor: Cortex
dag: Days after grafting
DE: Degree of esterification
e: Endodermis
f: Fibre
HG: Homogalacturonan
LR/lr: Lateral root(s)
p: Pericycle
PAS: Periodic acid-schiff
PBS: Phosphate-buffered saline
PCD: Programmed cell death
PMEs: Pectin methylesterases
rc: Root cap
sc: Scion
xy: Xylem vessels
